# Prenatal exposure to *Plasmodium falciparum* increases frequency and shortens time from birth to first clinical malaria episodes during the first two years of life: prospective birth cohort study

**DOI:** 10.1186/s12936-016-1417-0

**Published:** 2016-07-22

**Authors:** Boniphace Sylvester, Dinah B. Gasarasi, Said Aboud, Donath Tarimo, Siriel Massawe, Rose Mpembeni, Gote Swedberg

**Affiliations:** Department of Parasitology and Medical Entomology, School of Public Health and Social Sciences, Muhimbili University of Health and Allied Sciences, P.O.BOX 65001, Dar es Salaam, Tanzania; Department of Microbiology and Immunology, School of Medicine, Muhimbili University of Health and Allied Sciences, P.O.BOX 65001, Dar es Salaam, Tanzania; Department of Obstetrics and Gynaecology, School of Medicine, Muhimbili University of Health and Allied Sciences, P.O.BOX 65001, Dar es Salaam, Tanzania; Department of Community Medicine, School of Public Health and Social Sciences, Muhimbili University of Health and Allied Sciences, P.O.BOX 65001, Dar es Salaam, Tanzania; Department of Medical Biochemistry, Biomedical Centre, Uppsala University, Uppsala, Sweden

**Keywords:** Prenatal exposure, *Plasmodium falciparum*, Clinical malaria episode, Newborns, Susceptibility

## Abstract

**Background:**

Prenatal exposure to *Plasmodium falciparum* affects development of protective immunity and susceptibility to subsequent natural challenges with similar parasite antigens. However, the nature of these effects has not been fully elucidated. The aim of this study was to determine the effect of prenatal exposure to *P. falciparum* on susceptibility to natural malaria infection, with a focus on median time from birth to first clinical malaria episode and frequency of clinical malaria episodes in the first 2 years of life.

**Methods:**

A prospective birth cohort study was conducted in Rufiji district in Tanzania, between January 2013 and December 2015. Infants born to mothers with *P. falciparum* in the placenta at time of delivery were defined as exposed, and infants born to mothers without *P. falciparum* parasites in placenta were defined as unexposed. Placental infection was established by histological techniques. Out of 206 infants recruited, 41 were in utero exposed to *P. falciparum* and 165 infants were unexposed. All infants were monitored for onset of clinical malaria episodes in the first 2 years of life. The outcome measure was time from birth to first clinical malaria episode, defined by fever (≥37 °C) and microscopically determined parasitaemia. Median time to first clinical malaria episode between exposed and unexposed infants was assessed using Kaplan–Meier survival analysis and comparison was done by log rank. Association of clinical malaria episodes with prenatal exposure to *P. falciparum* was assessed by multivariate binary logistic regression. Comparative analysis of mean number of clinical malaria episodes between exposed and unexposed infants was done using independent sample *t* test.

**Results:**

The effect of prenatal exposure to *P. falciparum* infection on clinical malaria episodes was statistically significant (Odds Ratio of 4.79, 95 % CI 2.21–10.38, p < 0.01) when compared to other confounding factors. Median time from birth to first clinical malaria episode for exposed and unexposed infants was 32 weeks (95 % CI 30.88–33.12) and 37 weeks (95 % CI 35.25–38.75), respectively, and the difference was statistically significant (p = 0.003). The mean number of clinical malaria episodes in exposed and unexposed infants was 0.51 and 0.30 episodes/infant, respectively, and the difference was statistically significant (p = 0.038).

**Conclusions:**

Prenatal exposure to *P. falciparum* shortens time from birth to first clinical malaria episode and increases frequency of clinical malaria episodes in the first 2 years of life.

## Background

Sub-Saharan Africa still faces high rates of infant mortality due to *Plasmodium falciparum* infections, despite strategies put in place for the control of malaria [1]. In Tanzania, where this study was conducted, more than 80 % of the malaria infections are linked to *P. falciparum* [2]. It has been demonstrated that malaria due to *P. falciparum* during pregnancy may affect newborn susceptibility to subsequent malaria infections, and that infants born to mothers with placental malaria are exposed in utero to *P. falciparum* antigens [3–7]. The effect of early exposure to *P. falciparum* antigen on development of infant protective immunity has been studied and in utero exposed infants have been reported to have limited protective immune response to subsequent natural challenges with the same parasite antigen [8–10]. Epidemiological studies in some endemic areas have consistently demonstrated differences between children born to mothers with placental malaria (pm+) and those born to mothers without placental malaria (pm−).

A study conducted in Cameroon demonstrated that the prevalence of malaria parasitaemia in infants aged four to 6 months was higher in those born to pm+ mothers compared to the controls [11], while a study in Malawi demonstrated that the risk of mortality due to malaria was higher in infants born to pm+ mothers compared to those born to pm− mothers [12]. Although it has been established that prenatal exposure to malaria parasites influences the subsequent susceptibility of infants to malaria infection, there are other factors that may influence susceptibility to early malaria infections. The identified factors include but not limited to age of the mother, use of intermittent preventive treatment during pregnancy, gravidity and season of birth may influence the degree of exposure to malaria infection [13]. The dearth of information on the compounded effect of other factors and prenatal sensitization to the parasite antigens and their effects on subsequent susceptibility to the natural challenge of *P. falciparum* infection indicate that there remains a need to characterize this phenomenon.

This study was designed to systematically and prospectively follow up a birth cohort of infants for a period of 2 years, to delineate the effects of prenatal exposure to *P. falciparum* antigens and other factors (gravidity, season of birth, age of the mother, and infant birth weight) that could influence susceptibility, with respect to frequency and time from birth to first clinical malaria episode in infants born to pm+ and pm− mothers.

## Methods

### Study area

Four public health facilities (Utete, Mohoro Ikwiriri and Kibiti) located in Rufiji District, Coast Region in Tanzania were involved in this study. The area is low-lying, below 500 m above sea level and most of its surface area lies within the fertile flood plain of Rufiji River. Rufiji district typically experiences a long rainy season between February and May and a shorter, less intense one from October to December. Annual rainfall ranges between 800 and 1000 mm in the Rufiji River basin and malaria transmission occurs all year round.

### Study design, study population and recruitment of research participants

This was a prospective birth cohort study which included 206 mother infant pairs with infants aged 0–24 months. The criteria for recruitment of mothers included HIV seronegativity (pregnant mothers are routinely screened for HIV during prenatal visits in the study area) and confirmation to stay within the study area for a period of 2 years to facilitate follow up of infants to the age of 2 years. In order to maintain confidentiality, all study participants were assigned code numbers. The group assignment of the study participants was based on placental malaria status established by examination of placental tissues using histological techniques. Histological classification was not done in this study but the presence or absence of *P. falciparum* in the placenta at the time of delivery confirmed by histopathology was used to categorize the mothers and their infants. Mothers who had *P. falciparum* in their placenta at time of delivery confirmed by histopathology were defined as placental malaria positive (pm+) and their infants were defined as exposed to *P. falciparum*, while mothers who had no *P. falciparum* infection at time of delivery were defined as placental malaria negative (pm−) and their infants were defined as unexposed.

### Placental tissue collection, storage and histopathological examination

**Fig. 1:**
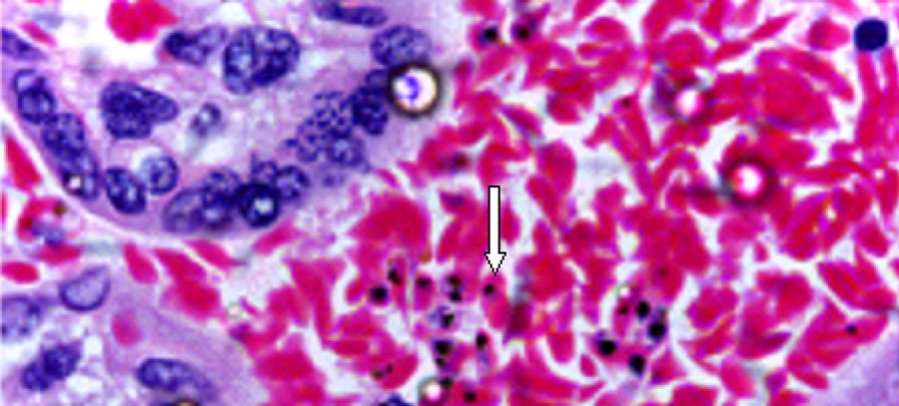
Demonstration of *Plasmodium falciparum* in intervillous erythrocytes in histological section of placenta stained using haematoxylin and eosin. 100× magnification. The section demonstrates *P. falciparum* in intervillous erythrocytes. An* arrow* is pointing at one of the parasitized (*P.falciparum*) intervillous red blood cell

In order to establish placental malaria infection histological samples were collected and processed. Briefly, following delivery, the maternal side of placenta was placed in an upright position and cleaned with sterile normal saline and a health paracentric area was then incised. Maternal placental tissue was examined using histopathologic techniques as previously described [14]. A full thickness of placental biopsy (8 cm^3^) was collected, by a trained and experienced nurse, from the maternal surface halfway between the placental edge and the insertion of the umbilical cord, within 4 hours of placental expulsion. The placental tissue was fixed in 10 % neutral-buffered formalin. Following fixation, placental tissue was trimmed and placed into histopathological cassettes, processed on an automated tissue processor overnight, embedded in paraffin wax, and then sectioned on a microtome to obtain 5-nm slices. The slices were placed in warm water bath at about 56 °C and transferred onto a microscope slide and left to dry and adhere to the slide. The placental sections were stained using haematoxylin and eosin and were then examined using light microscopic technique. Results were reported as placental malaria positive (Fig. 1) or placental malaria negative.

### Examination of placental blood specimen using a light microscope

**Fig. 2:**
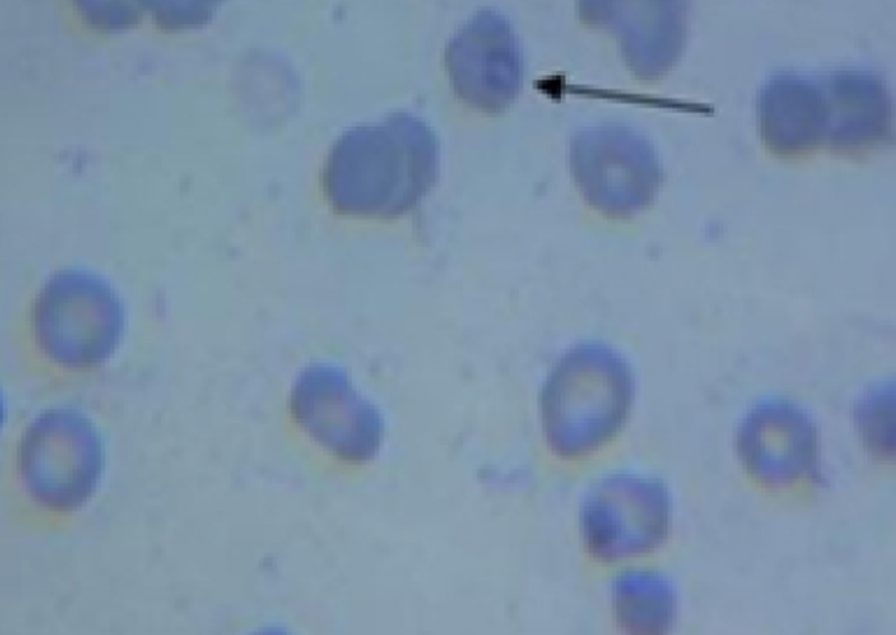
Thin blood smear prepared from placental blood showing *Plasmodium falciparum*. An* arrow* is pointing at* P.falciparum* in red blood cell

Using fresh placental blood specimen, thin smears were prepared, dried and fixed in methyl alcohol. Thick smears were not subjected to any fixative but dried and stained with 10 % working Giemsa for 10 min, washed, dried, and kept in slide boxes for microscopic examination. All smears were read independently by two trained laboratory technologists and discordant results on malaria species and parasite count were resolved by the Investigator. Results were reported as positive or negative for *P. falciparum* (Fig. 2). Documentation by photograph was done using Leica microsystem for future reference.

### Follow up of recruited infants and determination of clinical malaria episodes

Recruited infants were followed-up both at community and facility levels by trained research assistants. Scheduled visits were conducted regularly at 3 months intervals for a period of 2 years from birth while unscheduled visits were conducted whenever recruited infants had signs/symptoms of illness. Clinical examination was conducted by a trained study physician and clinical signs/symptoms were recorded on designated follow up questionnaire forms. Blood smears were collected for determination of clinical malaria infection using light microcopy technique. Clinical malaria was defined as fever (≥37.5 °C) with microscopically determined parasitaemia. Following the initial malaria episode, a new clinical malaria infection was defined as presence of asexual *P. falciparum* parasites in blood with fever (≥37.5 °C) after a period of 2 weeks or beyond from the previously treated clinical malaria episode. All Infants who were diagnosed of clinical malaria infection were treated within the routine system at the health facilities. In addition, infants who were diagnosed of non-malaria diseases were attended and provided with standard medical care during the entire study period.

Time to first clinical malaria episode was determined in all recruited infants in order to assess the vulnerability to malaria infection. Specific birth dates for the recruited infants and dates when they experienced clinical malaria episode for the first time were systematically recorded. Time elapsed from birth to diagnosis of clinical malaria episode was defined as the time to first clinical malaria episode. This was recorded for each recruited infant in weeks and the median time for each group was determined. Subsequent malaria episodes experienced by recruited infants were also recorded on the designed forms to determine the frequency of clinical malaria episodes during the first 24 months of life.

### Data management and analysis

Double-entry system was employed in data entry process. The data were cleaned and analysed using the Statistical Package for Social Sciences (SPSS), IBM version 20.0. Frequencies and cross-tabulations were used to summarize baseline socio-demographic characteristics between mothers with and without placental malaria (pm+ and pm−, respectively) and the association of prenatal exposure to *P. falciparum* with socio-demographic characteristics was determined using Chi square test. Comparison of mean number of clinical malaria episodes between exposed and unexposed infants was done using independent sample t test. The median times from birth to first clinical malaria episodes for infants born to mothers with placental malaria (exposed) and infants born to mothers without placental malaria (unexposed) were determined using Kaplan–Meier survival analysis and the comparison was carried out using Log rank test. Binary logistic regression analysis was used to assess the effect of prenatal exposure to *P. falciparum* on first clinical malaria episode. During the analysis, potential confounders were considered into the multivariate model and these included gravidity, birth weight, age of mother, and infant season of birth. Results were reported as odds ratio where applicable with 95 % confidence intervals. A ‘p’ value of <0.05 was considered to be significant.

## Results

### Socio-demographic characteristics of the study population

A total of 206 mother-infant pairs were included in the study. Of these, 41 infants were born to pm+ mothers (infants exposed in utero to *P. falciparum)* and 165 infants were born to pm− mothers (infants not exposed to *P. falciparum*). Table 1 summarizes the socio-demographic characteristics of the study population. Of 206 mothers recruited 66.9 % (n = 76) were primigravida and 63.1 % (n = 130) were multigravida. Out of 41 mothers who had placental malaria, 58.5 % (n = 24) were primigravida while 41.5 % (n = 17) were multigravida, and the difference was statistically significant (p < 0.01). There was no statistical significant difference between pm+ and pm− mothers on season of birth, level of education and age of mother. The use of insecticide treated bed nets (ITNs) between exposed infants and unexposed was similar (Table 1). The number of infants born with low birth weight was significantly higher in infants born to pm+ mothers compared to those born to pm− mothers, (Table 2) and the difference was statistically significant, p = 0.01.

**Table 1:** Social demographic characteristics of recruited mothers (n = 206)

Characteristics	Pm+ mothers n (%)	Pm− mothers n (%)	Chi square test p value
*Gravida*			
Primigravida	24 (58.5)	52 (31.5)	0.001
Multigravida	17 (41.5)	113 (68.5)	
*Marital status*			
single	8 (19.5)	24 (14.5)	0.3
Married	30 (73.2)	128 (77.6)	
Divorced	0	7 (4.2)	
Widow	0	3 (1.8)	
Cohabiting	3 (7.3)	3 (1.8)	
*Age of mother (years)*			
<18	3 (7.3)	13(7.9)	0.9
18–28	24 (58.5)	86 (52.1)	
29–39	8 (19.5)	43 (26.1)	
40–50	6 (14.6)	22 (13.3)	
Use of ITN	41 (100)	165 (100)	–
*Occupation*			
Peasant	34 (82.9)	135 (81.8)	0.3
Employed	1 (2.4)	4 (2.4)	
Large scale business	0	4 (2.4)	
Housewife	5 (12.2)	22 (13.3)	
Small scale business	1 (2.4)	0	
*Education level*			
Madras	8 (19.5)	36 (21.8)	0.8
Primary school	32 (78.0)	117 (70.9)	
Secondary school	1 (2.4)	10 (6.1)	

n(%) represents the numbers of mothers and their percentages in brackets

*ITN* insecticide treated bed nets*, pm*− placental malaria negative*, pm*+ placental malaria positive

**Table 2:** Social demographic characteristics of recruited infants (n = 206)

Characteristics	Infants born to pm+ mothers n (%)	Infants born to pm− mothers n (%)	Chi square test p value
*Infants birth weight (kg)*			
<2.5	10 (24.4)	16 (9.6)	0.01
≥2.5	31 (75.6)	149 (90.4)	
*Gender*			
Female	17 (41.5)	88 (53.3)	0.17
Male	24 (58.5)	77 (46.7)	
*Season of birth*			
Dry season	9 (30)	19 (11.5)	0.08
Wet season	32 (70)	146 (88.5)	
Use of ITN	41 (100)	165 (100)	–

(%) represents the numbers of infants and their percentages in brackets

*ITN* insecticide treated bed nets*, pm*− placental malaria negative*, pm*+ placental malaria positive

### Time to first clinical malaria episode and the mean number of clinical malaria episodes

**Fig. 3:**
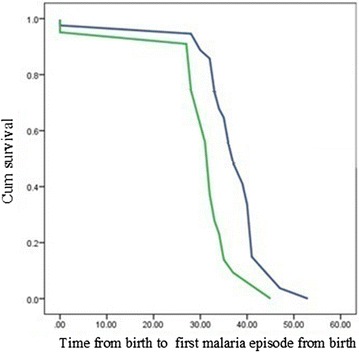
Kaplan Meier curve showing the survival of infants from birth to first clinical malaria episodes between exposed infants and unexposed infants. The* graph* portrays time at first clinical malaria episode in pm+ (*Bottom green line*) and pm− infants (*Top blue line*)

The median time from birth to first clinical malaria episode in exposed infants was 32 weeks (95 % CI 30.88–33.12), while in unexposed infants it was 37 weeks (95 % CI 35.25–38.75) and the difference in the median time was statistically significant (p = 0.003, Fig. 3). The mean number of clinical malaria episodes during the first 2 years of life, among infants born to pm+ mothers was 0.51 episodes/infant compared to those born to pm− mothers with 0.30 episodes/infant and the difference was statistically significant (p = 0.038).

### The effect of prenatal exposure to *Plasmodium falciparum* on clinical malaria episodes during the first 2 years of life

The effect of prenatal exposure to *P. falciparum* on clinical malaria episode was assessed using binary logistic regression. Important confounding factors included gravidity, season of birth, birth weight of infant, and age of mother. The statistical model has shown that fetal in utero exposure to *P. falciparum* is significantly associated with the vulnerability of infants to clinical malaria episodes (Odds Ratio 4.79, 95 % CI 2.21–10.38 p < 0.05) while other factors did not statistically influence the clinical malaria episodes in a multivariate analysis (Table 3).

**Table 3:** Effects of prenatal exposure to *Plasmodium falciparum*, gravidity, Infant birth weight, age of the mother at delivery, and season of birth to clinical malaria episodes: binary logistic regression

Factors	BLR-univariate analysis		BLR-multivariate analysis	
	OR (CI 95 %)	p value	AOR (CI 95 %)	p value
*In utero exposure to* *Plasmodium falciparum*				
Non-exposure to *P. falciparum* in utero	1 (–)	–	1 (–)	–
Exposure to *P. falciparum* in utero	4.632 (2.248–9.541)	p < 0.01	4.791 (2.21–10.38)	p < 0.01
*Gravidity of mothers*				
Primigravidity	1 (–)	–	1 (–)	–
Multigravidity	0.752 (0.4–1.415)	0.372	0.973 (0.475–1.990)	0.939
Infant birth weight				
Normal birth weight	1 (–)	–	1 (–)	–
Low birth weight	0.441 (0.189–1.031)	0.059	0.556 (0.209–1.477)	0.239
*Age of mothers (years)*				
40–50	1	–	1 (–)	–
<18	1.833 (0.626–5367)	0.269	2.120 (0.672–6.687)	0.20
18–28	1.100 (0.329–3.677)	0.877	1.196 (0.328–4.361)	0.786
29–39	1.615 (0.779–3.351)	0.198	2.013 (0.913–4.438)	0.083
*Infant season of birth*				
Dry season	1.0	–	1 (–)	–
Wet season	0.736 (0.311–1.741)	0.485	0.917(0.338–1.2.484)	0.864

Prenatal exposure to Plasmodium falciparum significantly associated with clinical malaria episodes in univariate and Multivariate analysis (p < 0.01)

*OR* Odds Ratio*, AOR* Adjusted Odds Ratio*, CI* Confidence interval*, BLR* Binary logistic regression

## Discussion

This prospective birth cohort study sought to delineate the effect of in utero exposure to *P. falciparum* infection on identified indicators of susceptibility, including time from birth to first clinical malaria episode and mean number of clinical malaria episodes. In addition, the effects of other factors that could confound the effect of in utero exposure to *P. falciparum* were also assessed. The median time from birth to first clinical malaria episode was significantly shorter in infants exposed in utero to *P. falciparum* infection than in unexposed infants. Clinical malaria infections in both exposed and unexposed infants were experienced beyond 6 months of age. This finding was corroborated by previous studies, which reported that malaria maternal antibodies are protective in the first 6 months of infant life [15–17]. The findings in this study of the occurrence of first clinical malaria episodes after 6 months of age in exposed and unexposed infants may indicate the possible protection of maternal antibodies as already demonstrated [18]. However, the current findings of the time from birth to first clinical malaria episode to be beyond 6 months following the waning of maternal antibodies are not consistent with the findings of a study done in Cameroon which indicated that maternal antibodies were not protective but markers of malaria infection [19].

Previous studies demonstrated that prenatal exposure to *P. falciparum* influenced the susceptibility to malaria infections by affecting the development of protective immunity against malaria infection through development of immuno-tolerance to the parasite antigen in subsequent malaria infections, rendering the exposed infants to be more susceptible to malaria infection than the unexposed infants [20]. The current study has demonstrated that infants born to placental malaria-positive mothers were more vulnerable and experienced the first clinical malaria episode much earlier in life after birth than infants born to mothers without placental malaria during the first 2 years of life. The other factors that could have influenced exposure to malaria infection, including level of education of the mother and use of ITNs, were similar in all recruited mothers and were therefore not considered as confounding. However, the factors which were considered in multivariate analysis have shown that, the age of the mother, season of birth, weight of infant at birth, and gravidity did not affect clinical malaria episodes in the study area. However, a similar study conducted in Ghana indicated that heterogeneity in exposure is likely to be the dominant factor influencing incidence of malaria in infancy [21]. The findings of the study in Ghana are corroborated by studies which have shown that the level of exposure to infective *Anopheles* mosquitoes, the environmental factors (which may influence mosquito density) and behavioral factors are important in malaria transmission and have a direct influence on exposure and susceptibility [22, 23].

Although the current study considered some of the factors which could confound the effect of prenatal exposure on susceptibility, it did not incorporate those factors that may have a direct influence on exposure to the parasite.

Lack of influence of the other exposure-related factors in this study is supported by the fact that, in the study area there was an ongoing anti-malaria campaign which involved distribution of ITNs at subsidized cost and emphasis was made on use of IPTp-SP for prevention of malaria during pregnancy as part of National Malaria Control Programme. The impact of this campaign in the study area was reflected in the general malaria prevalence which was reported to be 4.8 % [24]. However, the number of mothers with placental malaria seemed to remain high (n = 41) in the perceived context of the effects of placental malaria to the exposed infants.

In the present study, the mean number of clinical malaria episodes in the first 2 years of life was significantly higher in infants exposed to placental malaria than unexposed. These findings are corroborated by earlier studies, which demonstrated that infants born to pm+ mothers were more susceptible to *P. falciparum* infection [25–27].

However, a study done in Ghana indicated that the incidence of malaria in infants born to primigravida without placental malaria was significantly lower than that of infants born to primigravida with placental malaria but, there was no significant difference in the incidence of malaria among infants born to multigravida with or without placental malaria [21]. While the findings in infants born to primigravida with or without placental malaria are consistent with the findings in this study, it is notable that the findings in multigravida are not consistent with the finding in primigravida, putting into question the influence of prenatal exposure to the parasite antigen on susceptibility to malaria infection.

In this study, it was noted that a significantly higher proportion of primigravida were placental malaria-positive compared to multigravida (58.5 vs 31.5 %, respectively); hence, the majority of the infants exposed in utero to *P. falciparum* were born to primigravida compared to multigravida (24 mothers vs 17 mothers, respectively). The increased vulnerability of primigravida to placental malaria compared to other gravidities is corroborated by a study conducted in Angola [28], which demonstrated that primigravida were more susceptible to placental malaria due to demonstrated low levels of antibodies against chondroitin sulfate A; while these antibodies were higher in multigravida [29], which reflected a level of protection against placental malaria [30, 31]. It is possible that the demonstrated high levels of antibodies against chondroitin sulfate A in multigravida may have a synergistic effect on the protection conferred by maternal antibodies to infants born of multigravida. This conjecture is based on findings by a study which demonstrated that antibody responses to the N-terminal region of VAR 2CSA (*P. falciparum* antigen) during early pregnancy were associated with reduced risks for infection and low birth weight [31].

Although a direct influence of gravidity on susceptibility was not demonstrated in this study, one study conducted in the northern part of Tanzania showed that gravidity interacted with prenatal exposure to malaria and played a role in affecting susceptibility of infants to malaria infections [32]. This study, demonstrated that prenatal exposure to malaria significantly affected the susceptibility of infants to first clinical malaria episodes in a multivariate analysis, while gravidity did not significantly associate with time to first clinical malaria episode. It is hypothesized that this discrepancy may not be inherent to a basic biological or immunological factor because being primigravida is a risk to acquiring placental malaria, which, in essence, may predispose the infant to immune-tolerance and, hence, increasing susceptibility to subsequent malaria infection.

## Conclusion

Prenatal exposure to *P. falciparum* significantly influences vulnerability to malaria infection. Infants exposed in utero to parasite antigens succumb early to clinical malaria episodes and experience more frequent clinical malaria episodes than unexposed infants during the first 2 years of life.

## Abbreviations

AOR: adjusted odds ratio
IPTp: intermittent preventive treatment in pregnancy
ITN: insecticide-treated bed net
SP: sulfadoxine-pyrimethamine
Pm+: placental malaria positive
Pm−: placental malaria negative
